# Physical activity and sleep quality among Chinese college students: the serial mediating roles of anxiety and depression

**DOI:** 10.3389/fpsyg.2025.1635406

**Published:** 2025-09-15

**Authors:** Zhiwei Zhang, Zhaozhi Liu, Yongxia Lin, Mingkun Guo, Xiongzhuang Xu, Li Peng

**Affiliations:** College of Physical Education, Southwest University, Chongqing, China

**Keywords:** college students, physical activity, sleep, anxiety, depression

## Abstract

**Objective:**

Physical activity is a modifiable lifestyle factor that plays a significant role in promoting mental health and sleep quality. However, empirical studies exploring the mechanisms by which varying intensities of physical activity influence sleep quality—particularly the mediating roles of anxiety and depression—remain limited. This study aims to investigate the relationships between moderate- and vigorous-intensity physical activity and sleep quality among Chinese university students, with a focus on the serial mediating effects of anxiety and depression.

**Methods:**

A cross-sectional survey was conducted from October to November 2024, employing convenience sampling to recruit 5,803 undergraduate students from a university in Chongqing, China. Participants completed the International Physical Activity Questionnaire-Short Form (IPAQ-SF), the Pittsburgh Sleep Quality Index (PSQI), the Generalized Anxiety Disorder 7-item scale (GAD-7), and the Patient Health Questionnaire-9 (PHQ-9). Data were analyzed using a bootstrap-based serial mediation model.

**Results:**

(1) Compared to the low-intensity physical activity group, students engaging in moderate- and vigorous-intensity physical activity exhibited significantly lower levels of anxiety (*F* = 380.82, *p* < 0.01) and depression (*F* = 410.56, *p* < 0.01), along with better sleep quality (*F* = 594.25, *p* < 0.01). (2) Physical activity intensity was negatively correlated with sleep quality (*r* = −0.374, *p* < 0.01), anxiety (*r* = −0.299, *p* < 0.01), and depression (*r* = −0.306, *p* < 0.01); sleep quality was positively correlated with anxiety (*r* = 0.455, *p* < 0.01) and depression (*r* = 0.471, *p* < 0.01); anxiety and depression were highly positively correlated (*r* = 0.840, *p* < 0.01). (3) Serial mediation analysis indicated that moderate- and vigorous-intensity physical activity not only had direct effects on sleep quality but also exerted indirect effects through the independent and serial mediating roles of anxiety and depression.

**Conclusion:**

Among Chinese university students, different intensities of physical activity are significantly associated with anxiety, depressive symptoms, and sleep quality. Moderate- and vigorous-intensity physical activity levels are significantly correlated with lower anxiety and depression scores and better sleep quality. Serial mediation analysis further reveals that anxiety and depression have significant indirect effects in the relationship between physical activity intensity and sleep quality, indicating that the association between physical activity and sleep quality is partially influenced by the serial effects of anxiety and depression. Notably, vigorous-intensity physical activity demonstrates a stronger association with these psychological indicators and sleep quality.

## 1 Introduction

In recent years, sleep disturbances and mental health issues have become increasingly prevalent among Chinese university students, emerging as significant public health concerns that threaten both physical and psychological wellbeing (Zhu et al., 2024; Xiang et al., 2025). Surveys indicate that approximately 71% of university students habitually stay up late, and widespread sleep insufficiency and poor sleep quality are common phenomena (China Youth Daily, 2024). Persistent sleep problems not only impair physiological functioning but are also closely associated with negative emotional states such as anxiety and depression. These factors often interact and exacerbate one another, creating a self-perpetuating vicious cycle that is difficult to disrupt. This trend has garnered growing attention from both the public and the academic community. The university period represents a critical stage of psychological and physiological development. The accumulation of sleep difficulties and associated emotional distress during this time may exert long-lasting impacts on student's quality of life and their ability to adapt socially. Consequently, the identification of key determinants affecting sleep quality, and the formulation of effective intervention strategies, represent urgent challenges that demand scholarly attention. Enhancing sleep quality and alleviating psychological burden among university students is of paramount importance for fostering their holistic health and promoting both mental and physical development.

Physical activity (PA), characterized by its cost-effectiveness, convenience, and broad applicability, serves as a highly accessible intervention for improving sleep quality. A randomized controlled trial reported that engaging in regular exercise for a duration of 12 weeks produced short-term enhancement in sleep quality among adolescent girls (Hurdiel et al., 2017). Regular PA helps relax both body and mind and facilitates energy metabolism, thus improving sleep initiation and maintenance (Dzierzewski et al., 2014). PA also serves to regulate circadian body temperature rhythms, increases both the proportion and duration of deep (slow-wave) sleep, reduces rapid eye movement (REM) sleep, shortens sleep onset latency and nocturnal awakenings, and promotes physiological restoration and growth hormone secretion. Collectively, these effects enhance sleep architecture and overall sleep quality (Santos et al., 2007; Horne and Staff, 1983; Horne and Porter, 1975). Moreover, PA has been shown to stimulate melatonin secretion—especially in the context of moderate-to-vigorous intensity exercise—thereby further improving sleep quality (Hurdiel et al., 2017; Zisapel, 2018). Accordingly, we hypothesize that physical activity is positively associated with sleep quality among university students (H1).

Owing to academic, social, and career-related pressures, anxiety is highly prevalent among Chinese university students. While anxiety represents a normative emotional response, excessive or persistent anxiety that exceeds one's psychological tolerance may progress into affective disorders or somatic illnesses (Liu et al., 2024; Azoulay et al., 2013). Prior studies have demonstrated a dose–response relationship between physical activity and mental health in university student populations: higher levels of physical activity are associated with lower severity of anxiety symptoms (Tyson et al., 2010). Moreover, anxiety is recognized as a well-established risk factor for poor subjective sleep quality (Ahammed et al., 2021), and elevated anxiety has been linked to increased incidence of sleep disturbances (Grey et al., 2023). Based on this theoretical and empirical foundation, we propose the following hypothesis: anxiety mediates the relationship between physical activity and sleep quality (H2).

Depression represents another highly prevalent mental health concern among Chinese university students, characterized by persistent low mood, diminished interest or pleasure, and cognitive impairments that negatively affect both personal and academic functioning (Tao et al., 2024). Physical activity has been widely recognized as an effective non-pharmacological intervention for alleviating depressive symptoms (Kandola et al., 2019). Multiple meta-analyses have confirmed that regular physical activity can be as effective as antidepressant treatment in reducing depressive symptom severity (Liu et al., 2020; Chong et al., 2022). Beyond its direct benefits, depression is strongly intertwined with sleep quality: students with depression frequently experience sleep disturbances, while poor sleep, in turn, exacerbates depressive symptoms. Bacaro et al. (2024) emphasized that sleep quality serves as a critical indicator for evaluating the severity of depression and anxiety among students. High-quality sleep has the potential to mitigate depressive symptoms, whereas poor sleep heightens the risk of depression and worsens existing conditions (Cahuas et al., 2020; Ramos et al., 2021). Based on these theoretical and empirical insights, we hypothesize that depression mediates the relationship between physical activity and sleep quality (H3).

Anxiety and depression frequently co-occur; however, they differ in core symptomatology, diagnostic criteria, and treatment approaches. Three theoretical perspectives have been proposed regarding their relationship. The unidimensional model posits that anxiety and depression represent clinical manifestations of the same disease spectrum. The bidimensional model emphasizes their distinct pathophysiological mechanisms. The comorbidity model, in contrast, suggests that when anxiety and depression coexist, they constitute a unique clinical entity (Heimberg, 1996). Prior research has demonstrated that anxiety symptoms can predict the subsequent development of depressive symptoms (Cummings et al., 2014). As two of the most common mental health problems among university students, both anxiety and depression have been shown to be effectively alleviated through physical activity interventions (Petruzzello and Box, 2020; Chen et al., 2024). During and following physical activity, the brain releases neurotransmitters such as dopamine and hormones including endorphins, which generate short-term positive emotional states such as pleasure, vitality, and psychological flow (Zhang and Zhou, 2013). When students experience anxiety or depression, sleep quality typically declines. Conversely, poor sleep contributes to neuroendocrine dysregulation—manifested in chronic inflammation, disrupted melatonin secretion, and elevated glucocorticoid levels—which, in turn, increases the risk of anxiety and depression (Menke, 2019). Genetic studies have further indicated substantial overlap in the genetic determinants of insomnia, depression, and anxiety (Gehrman et al., 2011). Chen Baoxiang and colleagues reported that physical activity and sleep quality interact to influence symptoms of anxiety and depression in university students, with vigorous-intensity physical activity combined with high-quality sleep significantly reducing the risk of both conditions (Chen et al., 2024). Building upon these findings, the present study hypothesizes that physical activity may indirectly affect sleep quality by modulating levels of anxiety and depression, thereby forming a chain-mediated pathway (H4).

**Figure 1:**
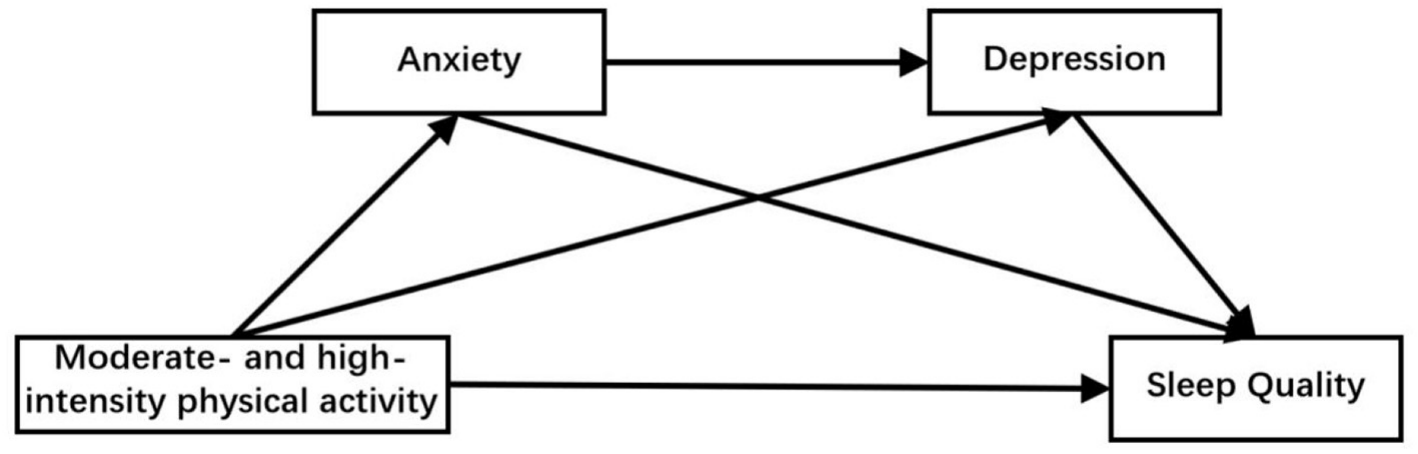
The serial mediation model of anxiety and depression between different levels of physical activity and sleep quality.

In summary, physical activity, as a modifiable lifestyle factor, plays a critical role in enhancing both mental health and sleep quality. Nevertheless, empirical evidence remains limited concerning the mechanisms by which varying intensities of physical activity influence sleep quality among university students, particularly with respect to the mediating roles of anxiety and depression. Accordingly, the present study focuses on a population of Chinese university students with three primary aims: (1) to examine differences in anxiety, depression, and sleep quality across different levels of physical activity intensity; (2) to explore the associations between physical activity and these psychological and behavioral variables; and (3) to test the mediating roles of anxiety and depression in the relationship between physical activity and sleep quality. This research seeks to provide a theoretical foundation for the development of targeted intervention strategies aimed at improving sleep quality in university populations. The proposed conceptual framework is presented in Figure 1.

## 2 Materials and methods

### 2.1 Participants

A convenience sampling strategy was adopted to recruit undergraduate students from a university in Chongqing, China, between October and November 2024. A total of 6,369 questionnaires were distributed. Following data collection, responses were rigorously screened according to the following exclusion criteria to ensure data validity: (1) incomplete responses or logically inconsistent answers; (2) response times judged to be insufficient for genuine engagement; (3) highly repetitive answer patterns across 10 or more consecutive items; and (4) questionnaires exhibiting excessively high response homogeneity. After applying these criteria, 5,803 valid questionnaires were retained, yielding an effective response rate of 91.11%.

Among the final sample, 2,493 participants were male (42.96%) and 3,310 were female (57.04%), with a mean age of 18.42 years (SD = 1.10). Prior to completing the survey, participants were thoroughly informed about the study's objectives, procedures, and confidentiality measures by trained research assistants. Written informed consent was obtained from all participants. The study was conducted in strict compliance with the ethical principles of the 1964 Helsinki Declaration and its later amendments. Ethical approval was granted by the Ethics Committee of Southwest University Hospital (Approval No. SWU-ETF-2023-07-17-011).

### 2.2 Research tools

#### 2.2.1 Physical activity

Physical activity was assessed using the International Physical Activity Questionnaire–Short Form (IPAQ-SF) (Lee et al., 2011), which comprises seven items. Participants reported the frequency (days per week) and duration (min per day) of walking, moderate-intensity, and vigorous-intensity physical activity performed for at least 10 min at a time over the preceding seven days. Each activity type was assigned a standardized Metabolic Equivalent of Task (MET) value as follows: walking = 3.3 METs, moderate-intensity activity = 4.0 METs, and vigorous-intensity activity = 8.0 METs. Weekly total energy expenditure was calculated using the formula: Total MET-min/week = Σ (frequency × duration × corresponding MET value). Based on international guidelines, participants were subsequently classified into low, moderate, or high physical activity levels.

#### 2.2.2 Sleep quality

Sleep quality was assessed using the Pittsburgh Sleep Quality Index (PSQI), a self-reported instrument developed by Buysse et al. (1989). The PSQI comprises 19 items distributed across seven components: subjective sleep quality, sleep latency, sleep duration, habitual sleep efficiency, sleep disturbances, use of sleep medication, and daytime dysfunction. Each component is scored on a 0–3 scale, yielding a global score ranging from 0 to 21. Higher scores indicate poorer sleep quality, with a global score greater than 5 suggesting significant sleep disturbances.

#### 2.2.3 Anxiety symptoms

Anxiety symptoms were assessed using the Generalized Anxiety Disorder 7-item Scale (GAD-7) (Toussaint et al., 2020). This self-report instrument consists of seven items evaluating the frequency of anxiety symptoms over the past 2 weeks, rated on a 4-point Likert scale ranging from 0 = “Not at all” to 3 = “Nearly every day.” An additional item assessing functional impairment over the same period is rated separately on a 4-point scale (“Not difficult at all,” “Somewhat difficult,” “Very difficult,” “Extremely difficult”) and is not included in the total score. The total score of the seven core items ranges from 0 to 21, with higher scores indicating greater severity of anxiety symptoms. Clinical cutoffs are as follows: 0–4 = no anxiety, 5–9 = mild anxiety, 10–14 = moderate anxiety, and ≥15 = severe anxiety. In the present study, the GAD-7 demonstrated excellent internal consistency (Cronbach's α = 0.92).

#### 2.2.4 Depressive symptoms

Depressive symptoms were assessed using the Patient Health Questionnaire-9 (PHQ-9) (Costantini et al., 2021), a self-report instrument based on the DSM-IV diagnostic criteria for depressive disorders. The PHQ-9 consists of nine items, each reflecting a core symptom of depression. It is widely used in both epidemiological research and clinical practice due to its brevity and ease of administration, with an ~ completion time of 5 min. Each item is rated on a 4-point Likert scale ranging from 0 = “Not at all” to 3 = “Nearly every day,” resulting in a total score ranging from 0 to 27. According to international clinical guidelines, total scores are interpreted as follows: 5–9 = mild depression, 10–14 = moderate depression, 15–19 = moderately severe depression, and ≥20 = severe depression. In the present study, the PHQ-9 demonstrated high internal consistency (Cronbach's α = 0.91).

### 2.3 Statistical analysis

Statistical analyses were performed using SPSS version 27.0. Normality tests were conducted for physical activity, anxiety, depression, and sleep quality. Variables that met the assumptions of normality were described using means and standard deviations (mean ± SD). Descriptive statistics, reliability analyses, multicollinearity diagnostics, and correlation analyses were subsequently conducted, followed by one-way ANOVA and Pearson correlation analyses. For the mediation analysis, the mediator variables (anxiety and depression) and the dependent variable (sleep quality) were treated as continuous variables. These continuous variables were standardized prior to analysis. The categorical independent variable, physical activity intensity (low, moderate, high), was dummy coded, with the low-intensity group serving as the reference category. Mediation effects were tested using the PROCESS macro (Model 6) in SPSS, examining the independent and chain-mediated roles of anxiety and depression in the relationship between physical activity and sleep quality. All statistical tests were two-tailed, and a significance level of *p* < 0.05 was adopted.

## 3 Results

### 3.1 Common method bias and multicollinearity assessment

Common method bias was examined using Harman's single-factor test across all items of the measurement instruments. Exploratory factor analysis revealed eight factors with eigenvalues greater than 1, with the first common factor accounting for 26.56% of the total variance, which is below the critical threshold of 40%. These results indicate that the measurement data were not substantially affected by common method bias.

Multicollinearity within the regression model was also evaluated. The variance inflation factors (VIF) for anxiety and depression were both 3.396, well below the commonly used threshold of 5 (or the more conservative threshold of 10), indicating no severe multicollinearity between the mediator variables. Therefore, the model estimates can be considered stable and reliable.

### 3.2 Differences in sleep quality, anxiety, and depression across physical activity intensity levels

The distribution of physical activity levels among participants was as follows: 15.49% engaged in low-intensity physical activity, 55.99% in moderate-intensity activity, and 28.16% in vigorous-intensity activity. The prevalence of anxiety symptoms was 23.78%, depression 22.76%, and poor sleep quality was reported by 15.54% of students.

A one-way analysis of variance (ANOVA) was conducted to examine the effects of physical activity intensity (low, moderate, high) on psychological outcomes. Significant group differences were observed for anxiety (*F* = 380.82, *p* < 0.01, partial η^2^ = 0.12), depression (*F* = 410.56, *p* < 0.01, partial η^2^ = 0.12), and sleep quality (*F* = 594.25, *p* < 0.01, partial η^2^ = 0.17). *Post hoc* analyses indicated that: (1) students in the moderate- (*M* = 2.11 ± 2.61) and vigorous-intensity (*M* = 1.54 ± 2.29) groups reported significantly lower anxiety levels than those in the low-intensity group (*M* = 4.81 ± 4.73); (2) depression scores were significantly lower in the vigorous-intensity group (*M* = 1.55 ± 2.53) compared with both the moderate- (*M* = 2.18 ± 2.87) and low-intensity groups (*M* = 5.40 ± 5.67); and (3) sleep quality was significantly better in the moderate- (*M* = 4.09 ± 2.28) and vigorous-intensity (*M* = 3.42 ± 1.98) groups relative to the low-intensity group (*M* = 6.48 ± 2.17). The detailed data are presented in Table 1.

**Table 1:** Analysis of differences in variables among different physical activity intensity groups ($\bar{x}$ ± s).

**Physical activity level**	***N* (%)**	**Anxiety**	**Depression**	**Sleep quality**
Low	899 (15.4%)	4.81 ± 4.73	5.40 ± 5.67	6.48 ± 2.17
Moderate	3,249 (55.99%)	2.11 ± 2.61	2.18 ± 2.87	4.09 ± 2.28
Vigorous	1,634 (28.16%)	1.54 ± 2.29	1.55 ± 2.53	3.42 ± 1.98
*F*	—	380.82^***^	410.56^***^	594.25^***^
Partial *η^2^*	—	0.12	0.12	0.17

^***^p < 0.001.

These results suggest that higher levels of physical activity—particularly vigorous-intensity activity—may confer benefits for mental health and sleep quality among university students.

### 3.3 Correlation analysis among physical activity, anxiety, depression, and sleep quality

As shown in Table 2, physical activity intensity was significantly negatively correlated with sleep quality, anxiety, and depression, indicating that higher levels of physical activity were associated with better sleep quality and lower levels of anxiety and depressive symptoms. Moreover, sleep quality was significantly positively correlated with both anxiety and depression, suggesting that poorer sleep quality is linked to greater psychological distress. A significant positive correlation was also observed between anxiety and depression, indicating that individuals with higher anxiety levels are more likely to exhibit more severe depressive symptoms.

**Table 2:** Correlation analysis of variables in Table 2 (*r*).

	**Physical activity**	**Sleep quality**	**Anxiety**	**Depression**
Physical activity	1	—	—	—
Sleep quality	−0.374^***^	1	—	—
Anxiety	−0.299^***^	0.455^***^	1	—
Depression	−0.306^***^	0.471^***^	0.840^***^	1

^***^p < 0.001.

### 3.4 Effects of physical activity, anxiety, and depression on sleep quality

As shown in Table 3, physical activity intensity was significantly negatively associated with anxiety, depression, and sleep problems, whereas anxiety was significantly positively associated with both depression and sleep problems, and depression was also significantly positively associated with sleep problems. These association patterns are consistent with the proposed hypotheses: higher levels of physical activity may be linked to lower anxiety and depression, which in turn are associated with better sleep quality; lower anxiety may relate to fewer depressive symptoms and improved sleep quality; and lower depression is associated with better sleep quality.

**Table 3:** Regression analysis of the serial mediation model involving anxiety and depression.

**Variables**	**Equation 1: (dependent variable: anxiety)**			**Equation 2: (dependent variable: depression)**			**Equation 3: (dependent variable: sleep quality)**		
	β	**SE**	**t**	β	**SE**	**t**	β	**SE**	**t**
Moderate-intensity physical activity	−0.83	0.04	−23.28^***^	−0.19	0.02	−8.90^***^	−0.62	0.03	−18.56^***^
vigorous-intensity physical activity	−0.97	0.04	−24.24^***^	−0.23	0.02	−8.87^***^	−0.82	0.04	−21.92^***^
Anxiety	—	—	—	0.81	0.01	107.37^***^	0.16	0.02	7.63^***^
Depression	—	—	—	—	—	—	0.23	0.02	11.41^***^
*R^2^*	0.13			0.71			0.31		
*F*	137.57^***^			2,026.91^***^			325.22^***^		

^***^p < 0.001.

Mediation effects were tested using a bootstrap approach with 5,000 resamples. As shown in Table 4, when moderate- and vigorous-intensity physical activity were treated as independent variables and anxiety and depression as mediators, the 95% confidence intervals for all three indirect pathways did not include zero, indicating significant mediation effects.

**Table 4:** Bootstrap analysis for testing the significance of mediation effects.

**Types of physical activity**	**Type of effect**	**Path**	**Effect size**	**SE/boot SE**	**Boot 95%CI**		**Percentage**
					**LLCI**	**ULCI**	
Moderate-intensity physical activity	Total effect		−0.96	0.03	−1.03	−0.89	100%
	Direct effect	Moderate-intensity physical activity → sleep quality	−0.62	0.03	−0.69	−0.56	64.58%
	Indirect effect	Moderate-intensity physical activity → anxiety → sleep quality	−0.13	0.02	−0.17	−0.09	13.54%
		Moderate-intensity physical activity → depression → sleep quality	−0.04	0.01	−0.06	−0.03	4.17%
		Moderate-intensity physical activity → anxiety → depression → sleep quality	−0.16	0.02	−0.20	−0.12	16.67%
Vigorous-intensity physical activity	Total effect		−1.21	0.04	−1.29	−1.13	100%
	Direct effect	Vigorous-intensity physical activity → sleep quality	−0.82	0.04	−0.89	−0.75	67.77%
	Indirect effect	Vigorous-intensity physical activity → anxiety → sleep quality	−0.15	0.02	−0.20	−0.11	12.40%
		Vigorous-intensity physical activity → depression → sleep quality	−0.06	0.01	−0.07	−0.04	4.96%
		Vigorous-intensity physical activity → anxiety → depression → sleep quality	−0.18	0.02	−0.22	−0.14	14.88%

^***^p < 0.001.

**Figure 2:**
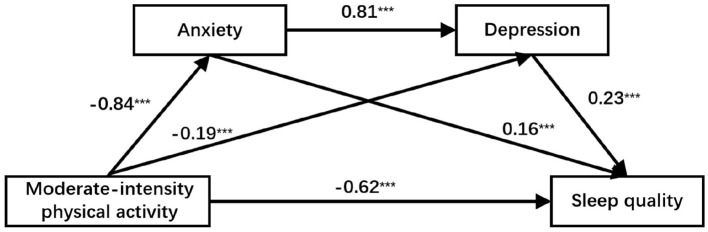
Chain mediation model of anxiety and depression between moderate-intensity physical activity and sleep quality.***p < 0.001.

**Figure 3:**
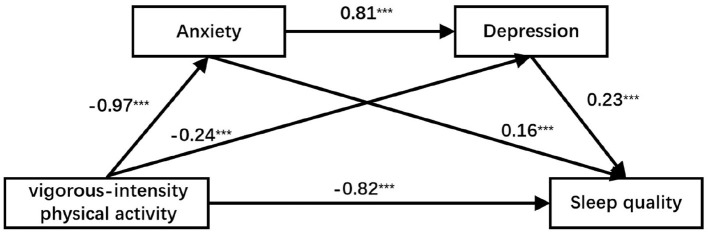
Chain mediation model of anxiety and depression between vigorous-intensity physical activity and sleep quality.***p < 0.001.

For moderate-intensity physical activity, the total effect on sleep quality was−0.96, with a direct effect of −0.62 (64.58%). The indirect effects occurred via three pathways: through anxiety (−0.13, 13.54%), through depression (−0.04, 4.17%), and through the anxiety → depression chain (−0.16, 16.77%). For vigorous-intensity physical activity, the total effect was −1.21, with a direct effect of −0.82 (67.77%). The corresponding indirect effects were: via anxiety −0.15 (12.40%), via depression −0.06 (4.96%), and via the anxiety → depression chain −0.18 (14.88%). The detailed structural equation models are presented in Figures 2, 3.

## 4 Discussion

### 4.1 Direct relationship between different intensities of physical activity and sleep quality among college students

This study identified a direct association between physical activity and sleep quality among college students, with higher levels of physical activity significantly linked to better sleep quality, thus supporting Hypothesis H1. A meta-analysis of prospective cohort studies has indicated that exercise is significantly associated with improvements in overall sleep quality (Kelley and Kelley, 2017). Existing research suggests that multiple forms of exercise—including aerobic exercise, resistance training, and mind–body practices such as Tai Chi—can enhance sleep quality. Aerobic exercise may promote neuroplasticity by modulating the expression of brain-derived neurotrophic factor (BDNF) genes, specifically through reducing methylation in promoter IV regions and increasing mRNA expression, which may serve as a key mechanism underlying its beneficial effects on sleep (Wrann et al., 2013). Whitworth et al. (2019) demonstrated that a 3-week vigorous-intensity resistance training intervention significantly improved sleep quality in patients experiencing post-traumatic stress disorder (PTSD)-related sleep disturbances. Mind–body exercises, including Tai Chi and yoga, combine focused attention, regulated breathing, and coordinated physical movements, thereby enhancing parasympathetic nervous system activity and facilitating emotional regulation, ultimately exerting positive effects on sleep quality.

In this study, the total effect of moderate-intensity physical activity on sleep quality was −0.96, with a direct effect of −0.62 (accounting for 65.58%), whereas vigorous-intensity physical activity had a total effect of −1.21 and a direct effect of −0.82 (accounting for 67.77%). These findings indicate that the negative association between vigorous-intensity physical activity and poor sleep quality is stronger than that observed for moderate-intensity activity. Existing research has shown that high-intensity interval training produces more pronounced improvements in sleep quality compared to moderate-intensity training (Jiménez-García et al., 2021). Experimental studies have also confirmed that participants in vigorous-intensity exercise groups exhibit greater improvements in sleep quality than those in low-intensity groups (Ji et al., 2022). Low-intensity physical activity may have limited effects on sleep quality due to insufficient stimulation of the cardiovascular and sympathetic nervous systems, as well as lower energy expenditure. A cohort study spanning 11.1 years found that low levels of physical activity exacerbate the adverse association between insufficient sleep and both all-cause and cause-specific mortality (Huang et al., 2022). Therefore, it is recommended that university students experiencing sleep problems prioritize moderate- and vigorous-intensity physical activity to achieve optimal sleep benefits.

### 4.2 Mediating role of anxiety and depression between different intensities of physical activity and sleep quality

Beyond the direct associations, this study found significant mediating effects of anxiety and depression in the relationship between physical activity and sleep quality within the mediation models. In the model with moderate-intensity physical activity as the independent variable, the indirect effects via anxiety and depression were −0.13 (13.54%) and −0.04 (4.17%), respectively. In the model with vigorous-intensity physical activity, the corresponding indirect effects were −0.15 (12.40%) and −0.06 (4.96%), respectively. These findings align with the patterns hypothesized in H2 and H3, indicating that the negative associations between physical activity and levels of anxiety and depression, together with the positive associations of anxiety and depression with poor sleep quality, constitute significant indirect pathways. Moreover, the effects through these pathways were stronger for vigorous-intensity than for moderate-intensity physical activity.

Physical activity was significantly negatively correlated with anxiety and depression, indicating that higher levels of physical activity are associated with lower anxiety and depressive symptoms. This finding is consistent with previous research. For instance, Gerber et al. (2014) reported that individuals engaging in vigorous-intensity exercise exhibited fewer mental health problems under conditions of high stress. Anxiety and depression are closely linked to the expression of brain-derived neurotrophic factor (BDNF), with insufficient BDNF expression potentially exacerbating negative emotional states. Physical exercise enhances the activity of antioxidant enzymes and increases BDNF levels; elevated BDNF, in turn, promotes neuroplasticity, neuronal growth, and differentiation, thereby effectively improving mood and alleviating anxiety and depression (Szuhany and Otto, 2020).

Furthermore, this study identified a significant negative correlation between anxiety, depression, and sleep quality. This finding aligns with Bacaro et al. (2024), who reported a bidirectional causal relationship between anxiety, depressive symptoms, and poor sleep quality. Previous research has demonstrated that depression can predict the onset of sleep disturbances (Zheng et al., 2018). Individuals with depression typically exhibit reduced brain-derived neurotrophic factor (BDNF) levels, and impaired sleep quality is also associated with alterations in BDNF concentrations (Monteiro et al., 2017). Additional studies have revealed a shared pathophysiological mechanism underlying insomnia and anxiety, namely hyperarousal resulting from dysregulation of neurotransmitter systems, including cholinergic and γ-aminobutyric acid (GABA) transmission (Clark and Watson, 1991). Genetic investigations further indicate substantial overlap in genetic influences on insomnia, depression, and anxiety (Gehrman et al., 2011). Moreover, hyperarousal and sleep deprivation can disrupt corticolimbic circuitry, thereby impairing emotional responses and regulatory processes.

### 4.3 The chain mediating role of anxiety and depression between different intensities of physical activity and sleep quality

Anxiety and depression are closely related, and they play a significant chain mediating role in the effect of different intensities of physical activity on sleep quality, confirming hypothesis H4. The chain mediation effect size for the path moderate-intensity physical activity → anxiety → depression → sleep quality was −0.16, while that for vigorous-intensity physical activity → anxiety → depression → sleep quality was −0.19.

Numerous studies have confirmed significant associations among physical activity levels, sleep quality, anxiety, and depression (Ghrouz et al., 2019; Chen et al., 2024). Ji et al. (2022) experimentally demonstrated that exercise influences anxiety, depression, and sleep quality in college students, with both exercise intensity and frequency affecting anxiety and depressive symptoms; however, exercise intensity exerts a greater effect than frequency. Furthermore, exercise intensity appears to be the primary factor in improving sleep quality, whereas exercise frequency does not significantly alter sleep outcomes. Some research indicates that individuals experiencing both sleep disturbances and mental health problems achieve greater benefits by increasing physical activity from moderate to moderate–high intensity, resulting in more pronounced improvements in both sleep quality and psychological wellbeing (Zhang et al., 2022). Patients with sleep disorders often exhibit physiological characteristics such as reduced oxidative stress levels and insufficient brain-derived neurotrophic factor (BDNF) expression. Under such conditions, increasing physical activity can substantially enhance sleep quality as well as alleviate anxiety and depressive symptoms.

The structural equation model not only revealed the associations among anxiety, depression, and sleep quality but also suggested that physical activity may be linked to college students sleep quality through psychological mechanisms. Specifically, higher levels of physical activity were associated with lower anxiety symptoms, which in turn were related to reduced depressive symptoms, ultimately jointly contributing to better sleep quality. Notably, the strength of these psychological associations varied with exercise intensity, with vigorous-intensity physical activity exhibiting significantly stronger effects than moderate-intensity activity. Additionally, depression functioned as a mediator in the relationship between anxiety and sleep quality—lower anxiety levels were associated with milder depressive symptoms, and this positive psychological state further corresponded to improved sleep quality. Over the long term, maintaining regular physical activity, particularly at vigorous intensity, may foster a beneficial pattern in which lower anxiety, lower depression, and better sleep quality coexist. These findings support the critical mediating roles of anxiety and depression in the relationship between physical activity and sleep quality.

## 5 Limitation

The findings of this study provide valuable empirical evidence and practical support for promoting the mental and physical health of Chinese university students, elucidating the relationships among different levels of physical activity, anxiety, depression, and sleep quality, as well as the chain-mediated associations between physical activity levels and sleep quality. Nevertheless, several limitations should be acknowledged. First, the cross-sectional design precludes causal inferences and cannot fully account for all potential confounding factors, such as academic stress, caffeine and alcohol intake, and body mass index; future studies could adopt longitudinal or experimental designs to further validate causal pathways. Second, this study primarily relied on self-report questionnaires, which may be subject to subjective bias; future research should integrate objective measures, such as accelerometers or sleep monitoring devices, to improve measurement accuracy. Third, convenience sampling was employed, potentially limiting sample representativeness and generalizability due to cultural, regional, and institutional influences; future studies should employ more diverse random sampling strategies to enhance generalizability and enable heterogeneity analyses. Furthermore, future research could explore the psychophysiological mechanisms through which physical activity influences sleep, such as autonomic nervous system function and hypothalamic-pituitary-adrenal axis regulation, providing theoretical support for developing precise, intensity-specific physical activity interventions.

## 6 Conclusion

Moderate- and vigorous-intensity physical activity were significantly associated with anxiety, depressive symptoms, and sleep quality among Chinese university students. Notably, vigorous-intensity physical activity demonstrated stronger associations with lower levels of anxiety and depression as well as better sleep quality. The chain-mediated model further revealed that anxiety and depression exert both independent and sequential mediating effects in the relationship between physical activity and sleep quality, indicating that physical activity may be linked to improved sleep quality through the alleviation of anxiety and depressive symptoms. These findings provide important empirical support for promoting mental health and sleep quality in university students through exercise interventions, highlighting vigorous-intensity physical activity as a priority recommendation for mitigating psychological and sleep-related problems in this population.

## Data Availability

The original contributions presented in the study are included in the article/supplementary material, further inquiries can be directed to the corresponding author.
